# Immunotherapy with low-dose IL-2 attenuates vascular injury in mice with diabetic and neovascular retinopathy by restoring the balance between Foxp3^+^ Tregs and CD8^+^ T cells

**DOI:** 10.1007/s00125-025-06412-8

**Published:** 2025-03-25

**Authors:** Devy Deliyanti, Varaporn Suphapimol, Amit Joglekar, Abhirup Jayasimhan, Jennifer L. Wilkinson-Berka

**Affiliations:** Department of Anatomy and Physiology, School of Biomedical Sciences, University of Melbourne, Parkville, VIC Australia

**Keywords:** Angiogenesis, CD8^+^ T cells, Diabetic retinopathy, IL-2, Oxygen-induced retinopathy, Regulatory T cells, Tregs

## Abstract

**Aims/hypothesis:**

Diabetic retinopathy features damage to the retinal microvasculature that causes vessels to leak and proliferate and can lead to vision loss and blindness. Inflammation contributes to the development of diabetic retinopathy, but little is known about the role of the adaptive immune system, including the benefits of augmenting the Forkhead box protein P3 (Foxp3) regulatory T cell (Treg) compartment. We aimed to determine whether treatment with low-dose IL-2 expands and activates Tregs and reduces CD8^+^ T cells in the retina, and attenuates retinal inflammation and vasculopathy in murine models of diabetic retinopathy and neovascular retinopathy.

**Methods:**

Mouse models of streptozocin-induced diabetes and oxygen-induced retinopathy (OIR) were administered low-dose IL-2 (25,000 U) or vehicle (sterile water) by i.p. injection. Reporter mice expressing Foxp3 as a red fluorescent protein (RFP) conjugate or CD8 as a green fluorescent protein (GFP) conjugate were used to evaluate Foxp3^+^ Tregs and CD8^+^ T cells, respectively, in blood, lymphoid organs and retina using flow cytometry or confocal microscopy. Vasculopathy and the expression of angiogenic and inflammatory factors were assessed in the retina.

**Results:**

Low-dose IL-2 significantly expanded CD4^+^CD25^+^Foxp3^+^ Tregs in the blood and spleen of mouse models of OIR and diabetes (1.4- to 1.9-fold increase, *p*<0.01). This expansion enhanced Treg functionality, increasing the expression of cytotoxic T-lymphocyte-associated protein4 (CTLA4), programmed cell death protein1 (PD1) and T-cell immunoreceptor with immunoglobulin and immunoreceptor tyrosine-based inhibitory motif (ITIM) domain (TIGIT), and increased the ratio of Tregs to CD8^+^ T cells. This was accompanied in the retina by a twofold increase in Foxp3^+^ Tregs (diabetes: 3.01 ± 0.41 vs 5.90 ± 1.25 cells per field, *p*<0.001; OIR: 4.41 ± 1.48 vs 10.05 ± 2.91 cells per field, *p*<0.001) and a reduction in CD8^+^ T cells (diabetes: 4.65 ± 0.58 vs 3.00 ± 0.81 cells per field, *p*<0.01; OIR: 5.51 ± 1.33 vs 3.17 ± 1.14 cells per field, *p*<0.01). Low-dose IL-2 reduced the levels of the potent inflammatory factors intercellular adhesion protein1 and TNF and the chemokine IFNγ-inducible protein10 (IP-10) in the retina. Importantly, low-dose IL-2 treatment effectively attenuated retinal vasculopathy, with marked reductions in acellular capillaries (diabetes: 0.48-fold decrease, *p*<0.001), neovascularisation (OIR: 0.68-fold decrease, *p*<0.01) and vascular leakage, and expression of vascular endothelial growth factor.

**Conclusions/interpretation:**

This study highlights the therapeutic potential of low-dose IL-2 to reduce retinal inflammation and severe vascular injury by boosting Tregs and reducing CD8^+^ T cells and inflammatory factors.

**Graphical Abstract:**

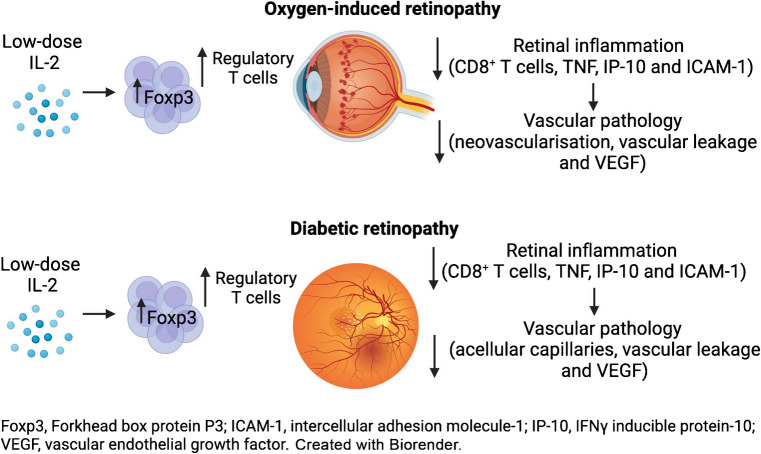

**Supplementary Information:**

The online version of this article (10.1007/s00125-025-06412-8) contains peer-reviewed but unedited supplementary material.


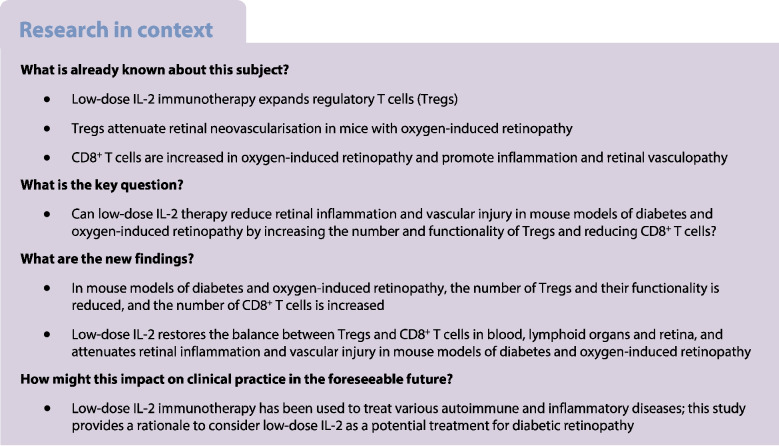


## Introduction

Diabetic retinopathy is a leading cause of blindness and vision impairment in the working-age population [1]. Damage to the retina’s microvasculature is a clinical hallmark of diabetic retinopathy and defines its progression from non-proliferative diabetic retinopathy to proliferative diabetic retinopathy, involving the growth of abnormal blood vessels. Diabetic retinopathy progresses as the microvasculature becomes devoid of pericytes and endothelial cells (acellular capillaries), resulting in tissue non-perfusion and ischaemia, breakdown of the blood–retinal barrier and neovascularisation. Inhibition of vascular endothelial growth factor (VEGF) using agents such as ranibizumab and aflibercept has revolutionised the treatment of diabetic retinopathy; however, their benefit is limited to those who have already lost some vision, they do not prevent diabetic retinopathy from advancing, they require repeated injections into the eye and, in many patients, their eyes respond insufficiently or not at all [2].

While many factors contribute to diabetic retinopathy, there is substantial evidence of a causal role for inflammation and the immune system. Retinal inflammation in diabetic retinopathy involves innate immune cells, including retinal microglia, which release proinflammatory factors such as TNF and IL-1b that injure the retina [3]. However, there is limited information about the protective or deleterious effects of adaptive immune cells, such as T cells. We have reported that the inflammation occurring in oxygen-induced retinopathy (OIR), a murine model of neovascular retinopathy with similarities to retinopathy of prematurity in preterm children, is influenced by regulatory T cells (Tregs) [4]. Tregs expressing the transcription factor Forkhead box protein P3 (Foxp3) exert potent anti-inflammatory actions by migrating from lymphoid tissues into injured tissues to suppress the damaging actions of effector cells, including CD8^+^ T cells [5]. We previously demonstrated that expanding the Treg compartment in peripheral lymphoid tissues led to trafficking of Tregs into the retina, presumably through a leaky blood–retinal barrier, and reduced inflammation and vasculopathy in OIR [4]. Recently, we reported that CD8^+^ T cells injure the retinal vasculature in OIR due to their release of inflammatory and cytotoxic factors, and their depletion protects against retinal inflammation and vasculopathy [6]. This information led to the hypothesis that similar events occur in diabetic retinopathy, and that immunotherapies that boost the abundance and functionality of Tregs will reduce CD8^+^ T cells, inflammation and vascular injury in the retina.

Our studies are focused on IL-2 as a potential therapeutic for diabetic retinopathy, as this cytokine binds with high affinity to the heterotrimeric IL-2 receptor, including the alpha subunit (CD25) on T cells and immune cells such as natural killer cells (NK cells) [7]. Tregs are highly sensitive to IL-2 compared with other immune cells due to their high levels of expression of the IL-2 receptor, which allows Tregs to sequester low levels of IL-2 that promotes their proliferation and limits the differentiation of effector cells such as CD8^+^ T cells [8, 9]. IL-2 was originally administered to patients at high doses as an immunotherapy for advanced melanoma and kidney cancer [10], but is associated with stimulation of all T and NK cells, which can lead to toxicity, including vascular leak syndrome and tissue damage [7, 11, 12]. In contrast, low-dose IL-2 increases Treg abundance and functionality, accompanied by a reduction in effector immune cells and organ protection, and is generally well tolerated [7, 13]. Preclinical studies have provided evidence for the use of low-dose IL-2 in clinical studies of systemic lupus erythematosus, type 1 diabetes and graft-vs-host disease [7, 14, 15].

The effect of low-dose IL-2 treatment has yet to be evaluated in diabetic retinopathy. Here, we used a mouse model of streptozocin-induced diabetic retinopathy to determine whether low-dose IL-2 increased the abundance and functionality of Tregs, as well as the Treg:CD8^+^ T cell ratio, in blood and lymphoid tissues. We speculated that this would be accompanied by an increase in Tregs and a reduction in CD8^+^ T cells in the retina, and the attenuation of retinal inflammation and vascular injury. As the mouse model of diabetes does not develop the retinal neovascularisation that occurs in proliferative diabetic retinopathy, the OIR mouse model was also studied.

## Methods

### Animals

The studies were approved by the University of Melbourne Ethics Committee (#10452) and adhered to the National Health and Medical Research Council of Australia’s guidelines for the care and use of animals in scientific research. Foxp3^rfp+/+^ and CD8^GFP+/−^ mice were bred in the University of Melbourne Bioresources Platform. Foxp3^RFP+/+^ mice are engineered to express a red fluorescent protein (RFP) in Foxp3^+^ cells [4]. CD8^GFP+/−^ mice (RRID: IMSR_JAX:008766, The Jackson Laboratory, USA; https://www.jax.org/strain/008766) express a green fluorescent protein (GFP) in CD8^+^ T cells. Female CD8^GFP+/−^ mice were mated with CD8^GFP−/−^ male mice to generate pregnant mice, and the heterozygous offspring were studied. All mice were housed at 21–22°C under a 12 h light/dark cycle, kept in group housing to promote social interaction, and had ad libitum access to food and water. All mice had a C57BL/6J background.

### Oxygen-induced retinopathy and diabetes models

**Fig. 1:**
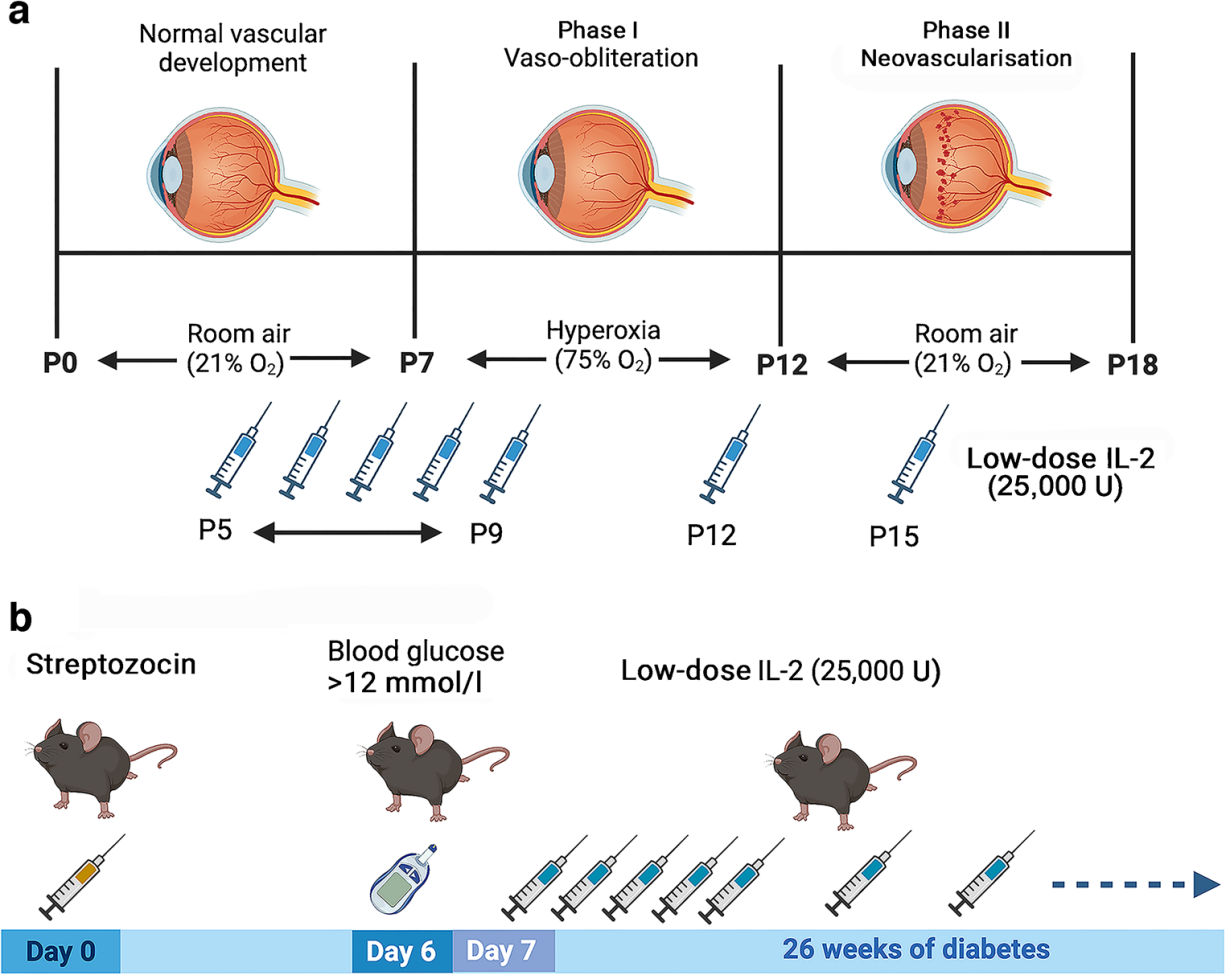
Experimental protocols and low-dose IL-2 treatment in mice. (**a**) Oxygen-induced retinopathy. Low-dose IL-2 was administered by daily i.p. injections for 5 days from P5 to P9 and then once every 3 days until P18. (**b**) Diabetic retinopathy. Seven days after the administration of streptozocin, diabetic mice were administered low-dose IL-2 by i.p. injection once per day for 5 days and then once every 3 days for 26 weeks. Created with BioRender

Induction of retinopathy was performed as described previously [4, 16, 17]. As sex does not influence OIR [18], both male and female mouse pups were studied. Pups and their nursing mothers were exposed to hyperoxia (75% oxygen) for 22 h per day between postnatal days 7 and 12 (P7 and P12) in specialised chambers with an atmosphere that was maintained using a Pro-ox 110 gas regulator (Biospherix) attached to medical-grade oxygen cylinders (BOC Gas) to allow retinal neovascularisation to develop. Age-matched control mice were housed in room air (21% oxygen) from birth until P18 (Fig. 1a). The body weight for each pup was recorded (ESM Table 1) and pups were killed at P18 using sodium pentobarbitone (150 mg/ml, Virbac). For studies of diabetes, all 6- to 8-week-old male Foxp3^RFP+/+^ and CD8^GFP+/−^ mice were assigned a number and then numbers were randomly selected by another researcher to receive streptozocin (50 mg/kg, Sigma) to induce diabetes or citric acid buffer (pH 4.5) by i.p. injection once each day for 5 consecutive days (Fig. 1b). Mice did not receive insulin. Female mice were not studied as they do not develop severe diabetic retinopathy. Blood glucose levels were measured 7 days after the first streptozocin injection and, if above 12 mmol/l, mice were considered diabetic. HbA_1c_ levels were measured using a Cobas B 101 analyser (Roche).

### Treatment with IL-2

IL-2 has a short half-life, being rapidly cleared from the circulation in <7 min [11]. Previous studies have determined a suitable schedule of administration to achieve an increased ratio of Tregs to T effector cells [19]. In mice, the induction phase to obtain the maximum increase in Tregs comprises a 5-day course of daily i.p. injections of IL-2. The maintenance phase to achieve sustained long-term increases in Tregs comprises i.p. injections once every 3 days [19].

As treatment with IL-2 can influence the abundance of effector immune cells such as NK cells, natural killer T cells (NKT cells) and CD8^+^ T cells, particularly when administered at high doses [20], the effect of low-dose IL-2 was compared with that of high-dose IL-2 in OIR studies. Litters of mice were randomised to be room air controls, OIR controls, OIR + low-dose IL-2 or OIR + high-dose IL-2, and studied at P18. Recombinant mouse IL-2 (#402-ML-500CF, R&D systems) in sterile water (pH 7.4) was administered as low-dose IL-2 (25,000 U) or high-dose IL-2 (250,000 U) via daily i.p. injections (50 µl) for 5 days from P5 to P9, then once every 3 days until P18, as described previously, in studies of diabetes [14, 20] (Fig. 1a). Control mice were administered vehicle (sterile water, 50 µl) using the same protocol. OIR mice were studied only if they showed consistent body weight gain in accordance with the criteria for OIR studies [4, 21]. In diabetes studies, 7 days after the administration of streptozocin, mice were randomised to receive low-dose IL-2 (25,000 U) or sterile water (controls) by i.p. injection (100 µl) once per day for 5 days and then once every 3 days for 26 weeks (Fig. 1b).

### Flow cytometry of blood, pooled lymph nodes and spleen

Samples were prepared as described previously [4]. Peripheral lymph nodes and spleens were mechanically disrupted and sieved through a 40 mm strainer to generate single-cell suspensions. For blood and spleen cells, 1 × RBC lysis buffer (#00–4333−57, eBiosciences) was used to remove erythrocytes. One million cells were incubated for 45 min at 4°C with the following antibody cocktails: (1) CD3 FITC (11–0032−82, RRID: AB_2572431, Thermo Fisher Scientific), CD4 BV786 (563727, RRID: AB_2728707, BD Biosciences), CD8 BV711 (563046, RRID: AB_2869451, BD Biosciences) and NK1.1 APC (550627, RRID: AB_398463, BD Biosciences) to detect NK, NKT and CD8^+^ T cells; and (2) CD3 APC-Cy7 (100222, RRID: AB_2242784, BioLegend), CD4 BV786 (563727, RRID: AB_2728707, BD Biosciences), CD8 BV711 (563046, RRID: AB_2869451, BD Biosciences), CD25 APC (561048, RRID: AB_10562035, BD Biosciences), cytotoxic T-lymphocyte-associated protein 4 (CTLA-4) PE (12–1522−82, RRID: AB_465879, Thermo Fisher Scientific), programmed cell death protein 1 (PD-1) PE-Cy7 (109109, BioLegend, RRID: AB_572016) and T cell immunoreceptor with immunoglobulin and immunoreceptor tyrosine-based inhibitory motif (ITIM) domain (TIGIT) BV421 (565270, RRID: AB_2688007, BD Biosciences) to detect Tregs and their activation markers. For intracellular staining of Foxp3^+^ Tregs, cells were further treated with fixation/permeabilisation solution (Thermo Fisher Scientific, 00–5523−00) according to the manufacturer’s protocols, and stained using Foxp3 antibody (Foxp3-PE-eF610, 61–5773−82, RRID:AB_2574624, eBiosciences). Cells were collected using a Fortessa X-20 flow cytometer (BD Biosciences) and analysed using FlowJo software (Tree Star version 10, BD Biosciences).

### Quantification of Foxp3^RFP+^ and CD8^GFP+^ T cells in retina

Eyes from Foxp3^RFP+/+^ and CD8^GFP+/−^ mice were collected and fixed in 4% paraformaldehyde in 0.1 M PBS for 30 min. The eyes were immediately flat-mounted and stained with DyLight 594 (1/100, DL-1207, VectorLabs) or FITC-conjugated isolectin GS-IB4 (1/100, L2895, Sigma) overnight at 4°C. RFP-expressing Tregs and GFP-expressing CD8^+^ T cells were imaged using a Nikon A1r confocal microscope at 20× magnification. Imaging was performed in the inner retina associated with neovascularisation, comprising the inner limiting membrane and ganglion cell layer. In diabetic mice, imaging was performed between the inner limiting membrane and outer nuclear layer. Twelve non-overlapping fields per retina were imaged and Foxp3^RFP+^ Tregs and CD8^+^ T cells counted and expressed as the number of cells per field.

### Retinal neovascularisation and vaso-obliteration

Quantification in OIR retinas was performed as described previously [4, 6]. The eyes were fixed in 4% paraformaldehyde in 0.1 M PBS for 30 min at room temperature. Retinal flat mounts were stained with FITC-conjugated isolectin GS-IB4 and imaged using a Zeiss Axio microscope attached to an AxioCam MRc camera (Carl Zeiss). Entire retinal montages were obtained using the tiling tool in the AxioObserver software (version 5.3, Carl Zeiss). ImageJ (https://imagej.net/ij/) was used to quantify neovascularisation and vaso-obliteration using the threshold and freehand tools, respectively.

### ELISA

In OIR studies, retinas were digested in 200 µl of TE-PER buffer (Invitrogen) containing a phosphatase/protease inhibitor cocktail (1/100, Thermo Fisher) using a bullet blender tissue homogeniser (Next Advance, New York, USA) at speed 9 for 5 min at 4°C. Protein lysates were centrifuged at 11,179 *g* for 10 min at 4°C, and supernatants were collected. Samples were run in duplicate for the ELISA assays using mouse albumin for vascular leakage (E-90AL, Immunology Consultants Laboratory, Portland, OR, USA) or mouse VEGF (DY493, R&D Systems) according to the manufacturer’s instructions. A limitation of the OIR studies is that vascular perfusion was not performed to exclude any contribution of albumin present in retinal blood vessels. In diabetic studies, vascular leakage was assessed by quantifying the albumin levels in the vitreous humour [17]. The total protein concentration of retinal homogenates and vitreous humour samples was measured using a Bradford assay (Bio-Rad). Albumin and VEGF levels were normalised to the total protein concentration.

### Retinal acellular capillaries in diabetes

Eyes were fixed with 4% paraformaldehyde in 0.1 M PBS for 1 h, and stored in 0.2 M Tris buffer (pH 8) at 4°C until use [22]. Retinas were digested in trypsin (3% in 0.2 M Tris buffer, pH 7.4) on a shaker at 37°C for 1 h, and incubated in Tris buffer on a rotator to remove loosely attached cells. The retinal vasculature was mounted on a slide and stained using periodic acid/Schiff reagent and haematoxylin. Twelve non-overlapping fields per retina were imaged at 200× using a Nikon UI microscope, and the number of acellular capillaries counted as described previously [22, 23].

### Real-time PCR

The methods used for real-time PCR are as described previously [4, 6]. Total RNA from a single retina was extracted using the RNeasy mini kit (Qiagen) and 500 ng RNA was subjected to DNase treatment (DNA-free kit, Ambion Life Technologies) and reverse transcription (Invitrogen). mRNA expression was normalised to that of 18s rRNA and is expressed as fold change using the comparative 2^−ΔΔCt^ method. The primer sequences for VEGF A, TNF and intercellular adhesion molecule1 (ICAM-1) were as published previously [4, 6]. The primer sequences for IP-10 were 5′-ATCATCCCTGCGAGCCTATCCT-3′ (forward primer) and 5′-GACCTTTTTTGGCTAAACGCTTTC-3′ (reverse primer).

### Statistical analyses

Statistical analyses were performed using GraphPad Prism version 9.0. Sample size calculations were based on prior studies and power analyses, aiming for 80% statistical power to detect significant effect size and minimise animal use. Data are presented as means ± SD. Normality was tested using Shapiro–Wilk tests. For normally distributed data, all groups were compared using one-way ANOVA, followed by the Holm–Šídák post hoc test. For non-normally distributed data, the Kruskal–Wallis test, followed by Dunn’s post hoc test, was used. Results were considered statistically significant at two-tailed *p* values less than 0.05. Experimental groups were randomised, and each data point represents an individual mouse or sample. Investigators were blinded to the experimental groups.

## Results

### Body weight and survival rates among OIR mice and diabetic mice

As expected, body weight was lower among OIR control mice than room air control mice. Treatment with low-dose IL-2 did not influence body weight or survival rates, but high-dose IL-2 slightly reduced body weight compared with OIR controls (ESM Table 1, ESM Fig. 1). Similarly, as expected, diabetic mice had lower body weights and higher blood glucose levels than non-diabetic mice (ESM Table 2). Low-dose IL-2 did not influence body weight, blood glucose levels (HbA_1c_) or survival rates of diabetic mice compared with diabetic control mice (ESM Table 2, ESM Fig. 1).

### Low-dose IL-2 expanded and activated peripheral Tregs and increased Tregs in OIR retinas

As IL-2 treatment can promote pathology due to the expansion of NK, NKT and CD8^+^ T cells [20], we evaluated their abundance in blood and lymphoid organs when retinal neovascularisation was established at P18. OIR had no effect on NK cells, although NKT cells were increased in pooled lymph nodes and with high-dose IL-2 treatment compared with room air controls (ESM Fig. 2). In blood, CD8^+^ T cells were increased in OIR controls and with high-dose IL-2 compared with room air controls (ESM Fig. 2). In the spleen of OIR mice, CD8^+^ T cells were increased by low-dose IL-2 and further increased by high-dose IL-2 compared with room air controls (ESM Fig. 2).

**Fig. 2:**
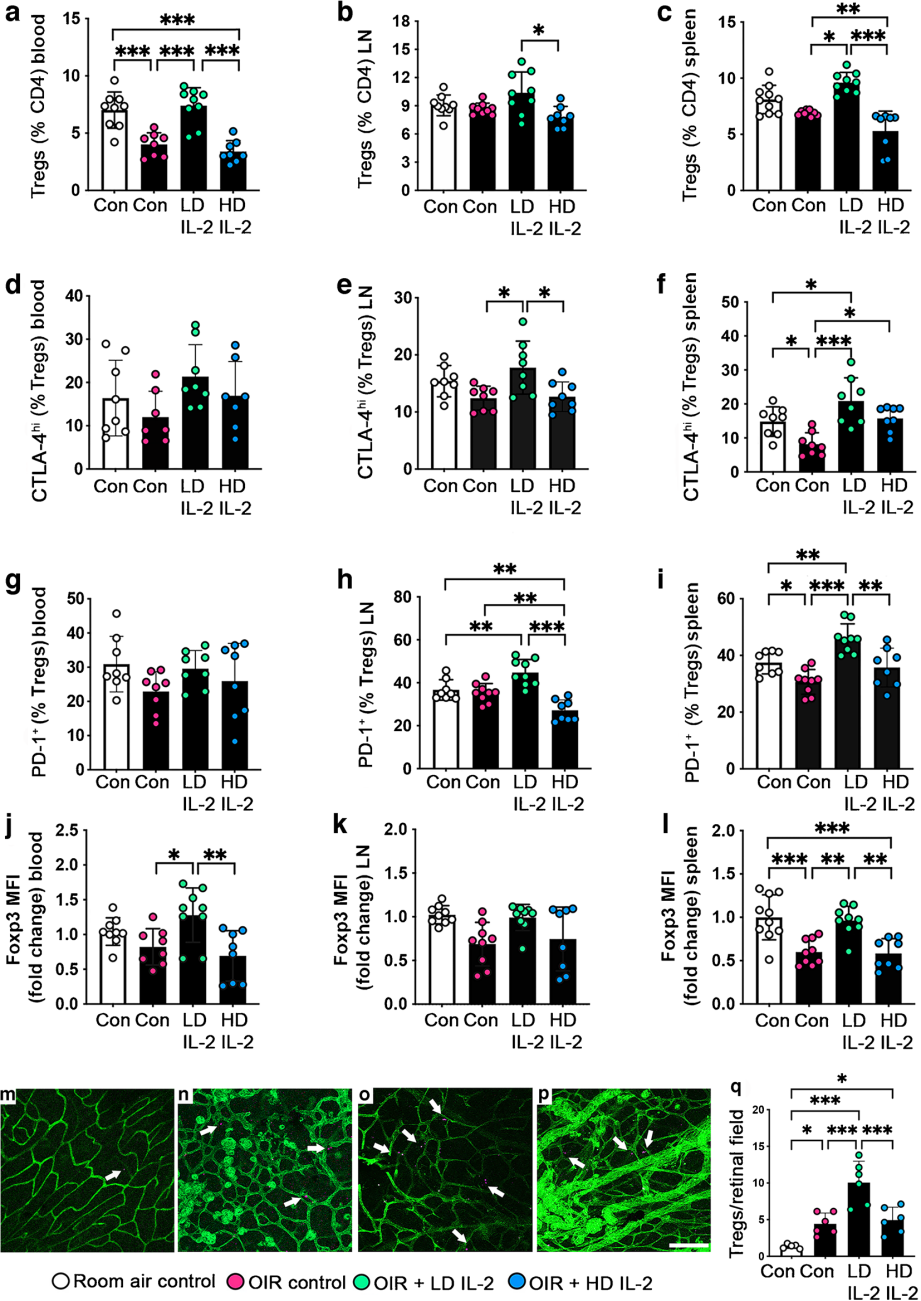
Low-dose IL-2 expanded Tregs in peripheral sites and retina of OIR mice at P18. Abundance of (**a**–**c**) CD4^+^CD25^+^Foxp3^+^ Tregs, (**d**–**f**) CTLA-4^hi^ and (**g**–**i**) PD-1^+^ Tregs, and (**j**–**l**) Foxp3 mean fluorescence intensity (MFI) levels in CD4^+^CD25^+^Foxp3^+^ Tregs in blood, pooled lymph nodes (LN) and spleen, measured by flow cytometry; *n=*7–10 mice per group with at least two independent experiments. (**m**–**p**) Representative flat mounts of retinas from Foxp3^RFP+/+^ mice, stained with FITC-conjugated isolectin B4 to show the vasculature (green): (**m**) room air control; (**n**) OIR control; (**o**) OIR + LD IL-2; (**p**) OIR + HD IL-2. Arrows indicate Foxp3^+^ Tregs, which were frequently found near areas of retinal neovascularisation in OIR controls and OIR + high-dose IL-2 mice. Scale bar = 100 µm. (**q**) Quantification of Tregs per field of inner retina (inner limiting membrane to ganglion cell layer) from Foxp3^RFP+/+^ mice; *n*=6–7 mice per group from two independent experiments. Con, controls; LD, low-dose; HD, high-dose. White bars are room air controls; black bars are OIR groups. All values are means ± SD. Data were analysed using one-way ANOVA and Holm–Šídák tests, except for the lymph node data in (**b**) and (**k**), which were analysed using the Kruskal–Wallis test followed by Dunn’s test for non-parametric comparisons. *p<0.05, **p<0.01, ***p<0.001

The abundance of Tregs in OIR controls was reduced in blood (Fig. 2a), and their suppressive function, comprising CTLA-4, PD-1 and Foxp3 (mean fluorescence intensity), was reduced in spleen compared with room air controls (Fig. 2f, i, l). In OIR mice, high-dose IL-2 did not restore Treg abundance in blood (Fig. 2a) or their suppressive function compared with OIR controls, except in terms of CTLA-4 expression in spleen (Fig. 2f). In contrast, low-dose IL-2 increased Treg abundance in the blood of OIR mice to room air control levels (Fig. 2a), increased CTLA-4 and PD-1 expression in spleen compared with room air and OIR controls (Fig. 2f, i), and increased the Foxp3 mean fluorescence intensity in spleen compared with OIR controls (Fig. 2i). To determine whether the increase in Tregs induced by low-dose IL-2 resulted in more Tregs in the retina specifically, we quantified Foxp3^+^ cells using Foxp3^RFP+/+^ mice. In OIR mice, Tregs were increased in OIR controls compared with room air controls (Fig. 2m, n, q), and low-dose but not high-dose IL-2 increased Foxp3^RFP+^ cells compared with room air and OIR controls (OIR control: 4.41 ± 1.48 vs OIR + low-dose IL-2: 10.05 ± 2.91 cells per field, *p*<0.001) (Fig. 2o–q) High-dose IL-2 slightly increased Tregs in OIR mice compared with room air controls, but not above OIR control levels (Fig. 2q).

### Low-dose IL-2 increased the peripheral Treg:CD8^+^ T cell ratio and reduced CD8^+^ T cells in OIR retinas

**Fig. 3:**
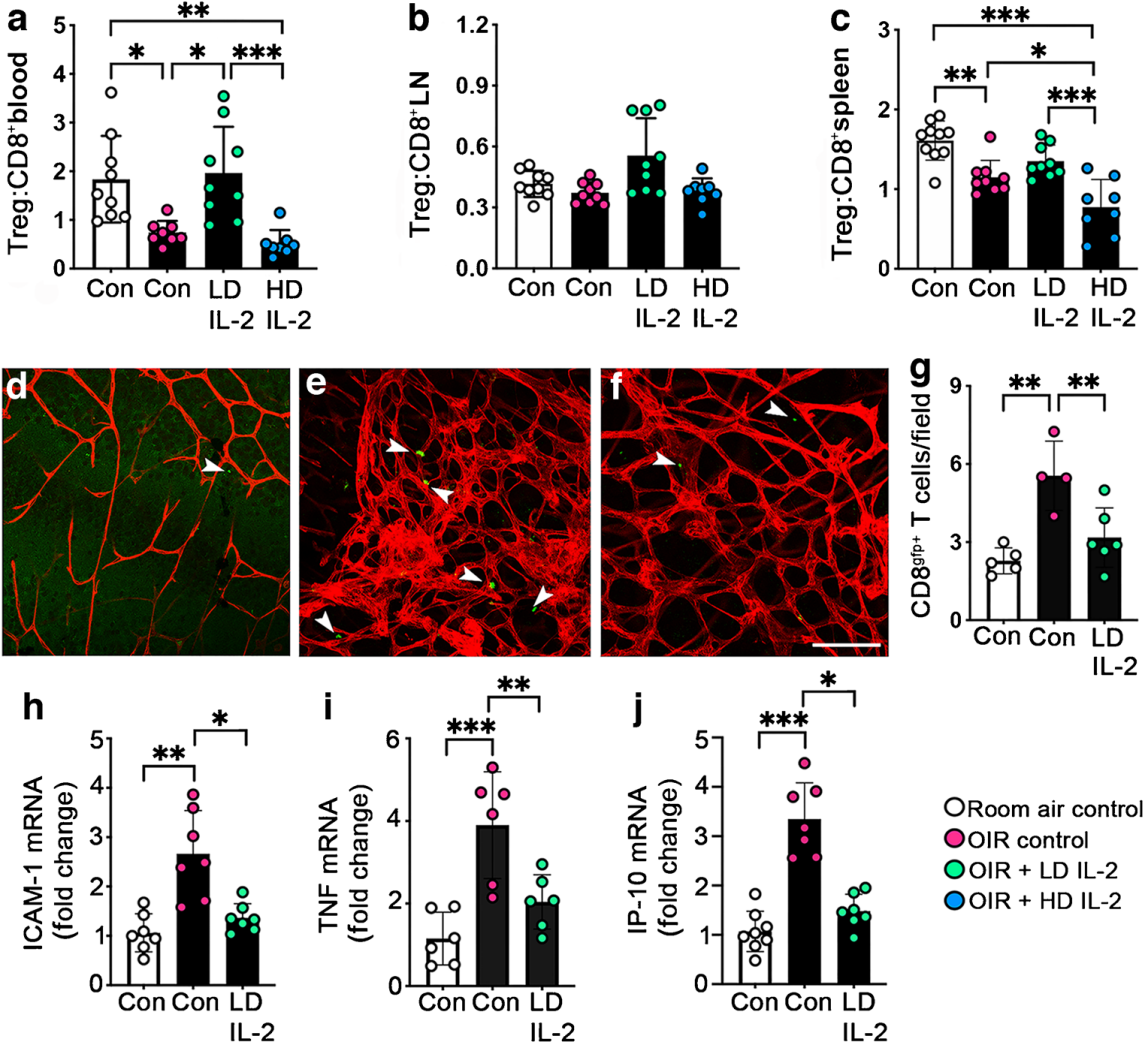
Low-dose IL-2 restored the Treg:CD8^+^ T cell ratio and reduced CD8^+^ T cells in retinas of OIR mice at P18. (**a**–**c**) Tregs:CD8^+^ T cells ratio in (**a**) blood, (**b**) pooled lymph nodes (LN) and (**c**) spleen measured by flow cytometry; *n*=8–10 mice per group from two or three independent experiments. (**d**–**f**) Representative flat mounts of retinas from CD8^GFP+/−^ mice, stained with DyLight 594/isolectin-conjugated isolectin B4 to show the vasculature (red). Arrowheads indicate CD8^+^ T cells (green): (**d**) room air control; (**e**) OIR control; (**f**) OIR + low-dose IL-2. Scale bar = 100 µm. (**g**) Quantification of CD8^+^ T cells per field of inner retina (inner limiting membrane to ganglion cell layer) from CD8^GFP+/−^ mice; *n*=4–6 mice per group. (**h**–**j**) Fold change in mRNA levels in retinas for (**h**) ICAM-1, (**i**) TNF and (**j**) IP-10 compared with room air controls; *n*=6–8 mice per group. Con, controls; LD, low-dose; HD, high-dose. White bars are room air controls; black bars are OIR groups. All values are means ± SD. All data were analysed by the Kruskal–Wallis test followed by Dunn’s test, except for data in (**c**) and (**g**), which were analysed using one-way ANOVA and the Holm–Šídák test for parametric comparisons. *p<0.05, **p<0.01, ***p<0.001

A flow cytometric analysis revealed that, in OIR controls, the Treg:CD8^+^ T cell ratio was reduced in blood and spleen, but not in pooled lymph nodes, compared with room air controls at P18 (Fig. 3a–c). In OIR mice, low-dose IL-2 restored the Treg:CD8 T cell ratio in blood but not in spleen (Fig. 3a–c). High-dose IL-2 did not increase the Treg:CD8 T cell ratio in blood or pooled lymph nodes in OIR mice compared with room air and OIR controls, and reduced the ratio in spleen compared with OIR controls (Fig. 3a–c). Our previous study demonstrated that CD8^+^ T cells are recruited into the retina during OIR [6]. Here, the increase in CD8^+^ T cells in the retina was reduced by low-dose IL-2 in OIR CD8^GFP+/−^ mice (OIR control: 5.51 ± 1.33 vs OIR + low-dose IL-2: 3.17 ± 1.14 cells per field, *p*<0.01) (Fig. 3d–g). As high-dose IL-2 did not increase the Treg:CD8^+^ T cell ratio in blood and lymphoid tissues, this treatment was not further evaluated. The inflammatory mediators ICAM-1 and TNF contribute to retinal vasculopathy [24, 25] and IP-10 is involved in CD8^+^ T cell recruitment into the retina [6]. ICAM-1, TNF and IP-10 expression was increased in the retinas of OIR controls compared with room air controls, and reduced in OIR mice treated with low-dose IL-2 (Fig. 3h–j).

### Low-dose IL-2 reduced retinal vascular pathology in OIR mice

**Fig. 4:**
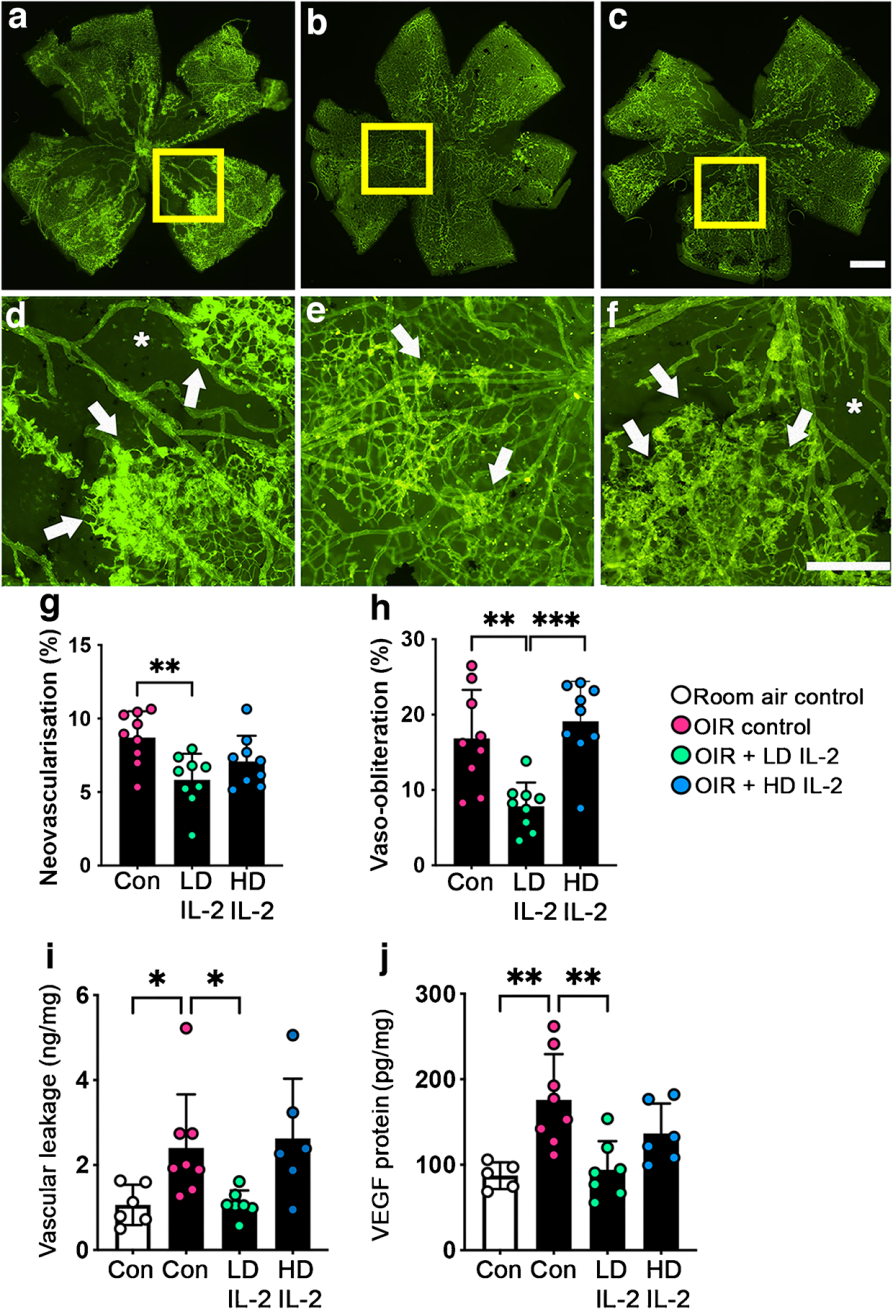
Low-dose IL-2 but not high-dose IL-2 attenuated retinal vasculopathy in OIR mice at P18. (**a**–**f**) Representative flat mounts of retinas from Foxp3^RFP+/+^ mice, stained using FITC-conjugated isolectin B4 to show the vasculature (green). The regions indicated by the yellow boxes in the upper panels are presented at higher magnification in the lower panels. Neovascularisation is indicated by arrows and vaso-obliteration is indicated by asterisks. Scale bars = 0.125 mm. (**g**, **h**) Quantification of retinal neovascularisation (**g**) and vaso-obliteration (**h**); *n*=9 mice per group from three independent experiments. (**i**, **j**) Vascular leakage in retina measured by an albumin ELISA (**i**) and VEGF protein levels in retina measured by ELISA (**j**); *n*=5–8 mice per group. Con, controls; LD, low-dose; HD, high-dose. White bars are room air controls; black bars are OIR groups. All values are means ± SD. All data were analysed by one-way ANOVA followed by the Holm–Šídák test, except for the data in (**j**), which were analysed using the Kruskal–Wallis test followed by Dunn’s test. *p<0.05, **p<0.01, ***p<0.001

As expected, OIR controls developed retinal neovascularisation and vaso-obliteration (Fig. 4a–h). In OIR mice, low-dose but not high-dose IL-2 reduced neovascularisation (0.68-fold decrease) and vaso-obliteration compared with OIR controls (Fig. 4a–h). In OIR controls, vascular leakage and VEGF protein levels in the retina were increased compared with room air controls. Low-dose IL-2 but not high-dose IL-2 reduced vascular leakage and VEGF protein levels compared with OIR controls (Fig. 4i, j).

### Low-dose IL-2 expanded and activated peripheral Tregs and increased Tregs in the retinas of mice with diabetic retinopathy

**Fig. 5:**
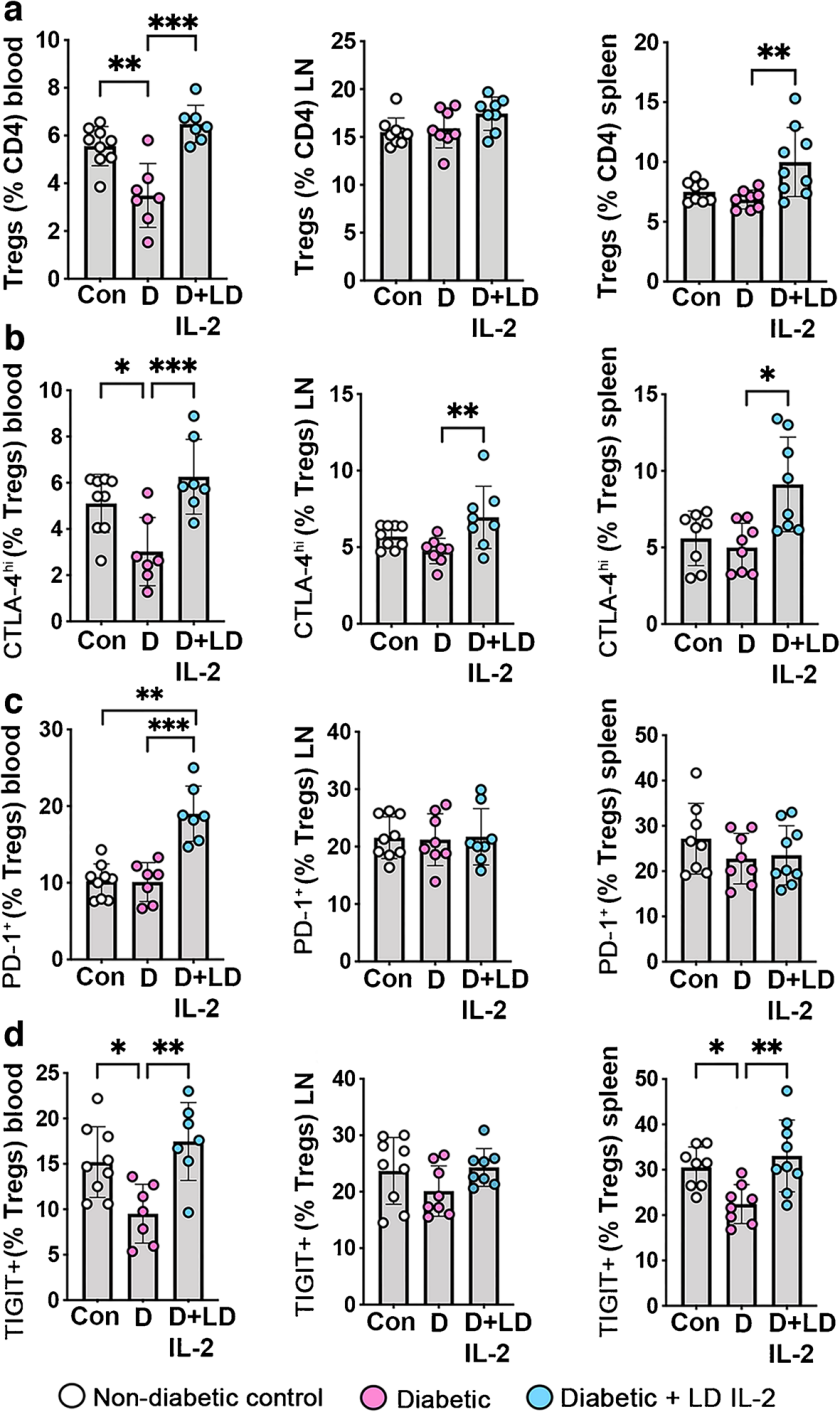
Low-dose IL-2 increased the number of Tregs in the blood and lymphoid tissues of mice with diabetic retinopathy for 26 weeks. (**a**–**d**) Abundance of (**a**) CD4^+^CD25^+^Foxp3^+^ Tregs and (**b**) CTLA-4^hi^, (**c**) PD-1^+^ and (**d**) TIGIT^+^ Tregs in blood, pooled lymph nodes (LN) and spleen, measured by flow cytometry; *n*=7–9 mice per group with at least two independent experiments. Con, non-diabetic control; D, diabetic mice; LD, low-dose. All values are means ± SD. All data were analysed by one-way ANOVA followed by the Holm–Šídák test, except spleen data in (**a**) and (**b**), which were analysed using Kruskal–Wallis and Dunn’s tests for non-parametric comparisons. *p<0.05, **p<0.01, ***p<0.001

**Fig. 6:**
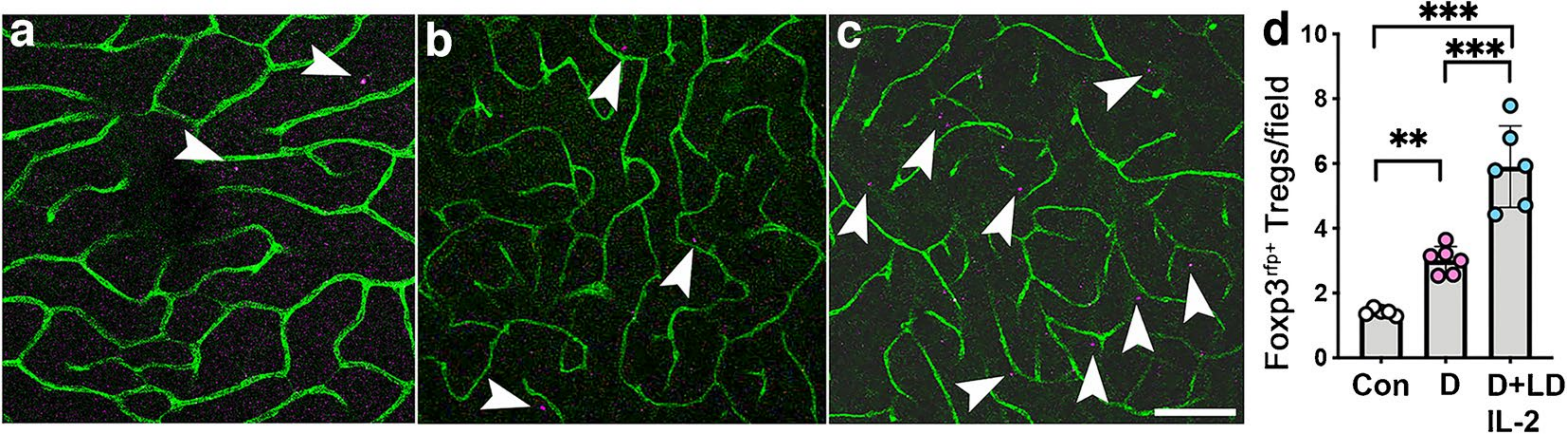
Low-dose IL-2 increased the number of Foxp3^+^ Tregs in the retinas of mice with diabetic retinopathy for 26 weeks. (**a**–**c**) Representative flat mounts of retina from Foxp3^RFP+/+^ mice, stained using FITC-conjugated isolectin B4 to highlight the vasculature (green): (**a**) non-diabetic control; (**b**) diabetic mice; (**c**) diabetic mice treated with LD IL-2. Arrowheads indicate Foxp3^+^ cells. Scale bar = 100 µm. (**d**) Quantification of Tregs per field of retina (inner limiting membrane to outer nuclear layer); *n*=6–7 mice per group from two independent experiments. Con, non-diabetic control; D, diabetic mice; LD, low-dose. All values are means ± SD. Data were analysed by one-way ANOVA followed by the Holm–Šídák test. **p<0.01, ***p<0.001

As for OIR studies, we evaluated the abundance of NK, NKT and CD8^+^ T cells in mice with diabetic retinopathy. Diabetes did not influence the abundance of these cell types in blood, pooled lymph nodes and spleen compared with non-diabetic controls (ESM Fig. 3). Low-dose IL-2 had minimal effects, increasing NK cells in pooled lymph nodes compared with non-diabetic controls and reducing CD8^+^ T cells in the blood compared with untreated diabetic mice (ESM Fig. 3). With respect to Tregs, their number was reduced in the blood of untreated diabetic mice compared with non-diabetic controls, and increased in diabetic mice treated with low-dose IL-2 (Fig. 5a). In spleen, low-dose IL-2 increased Tregs compared with non-diabetic and diabetic controls (Fig. 5a). We also examined whether low-dose IL-2 increased Treg functionality. In untreated diabetic mice, there were fewer functional Tregs, with the levels of CLTA-4^hi^ Tregs in blood and TIGIT^+^ Tregs in blood and spleen reduced compared with non-diabetic controls. In diabetic mice, low-dose IL-2 increased CLTA-4^hi^ Tregs in all sites studied and increased PD-1^+^ Tregs in blood and TIGIT^+^ Tregs in blood and spleen, compared with untreated diabetic mice (Fig. 5b–d). We next evaluated whether low-dose IL-2 increased Foxp3^+^ Tregs in retinas using Foxp3^RFP+/−^ mice. In untreated diabetic mice, Foxp3^+^ cells were increased in retinal tissue compared with non-diabetic controls (Fig. 6). In diabetic mice, low-dose IL-2 increased Foxp3^+^ cells in the retina compared with non-diabetic and diabetic controls (untreated diabetes: 3.01 ± 0.41 vs diabetes + low-dose Il-2: 5.90 ± 1.25 cells per field, *p*<0.001) (Fig. 6).

### Low-dose IL-2 increased the Treg:CD8^+^ T cell ratio and reduced retinal inflammation in diabetic retinopathy

**Fig. 7:**
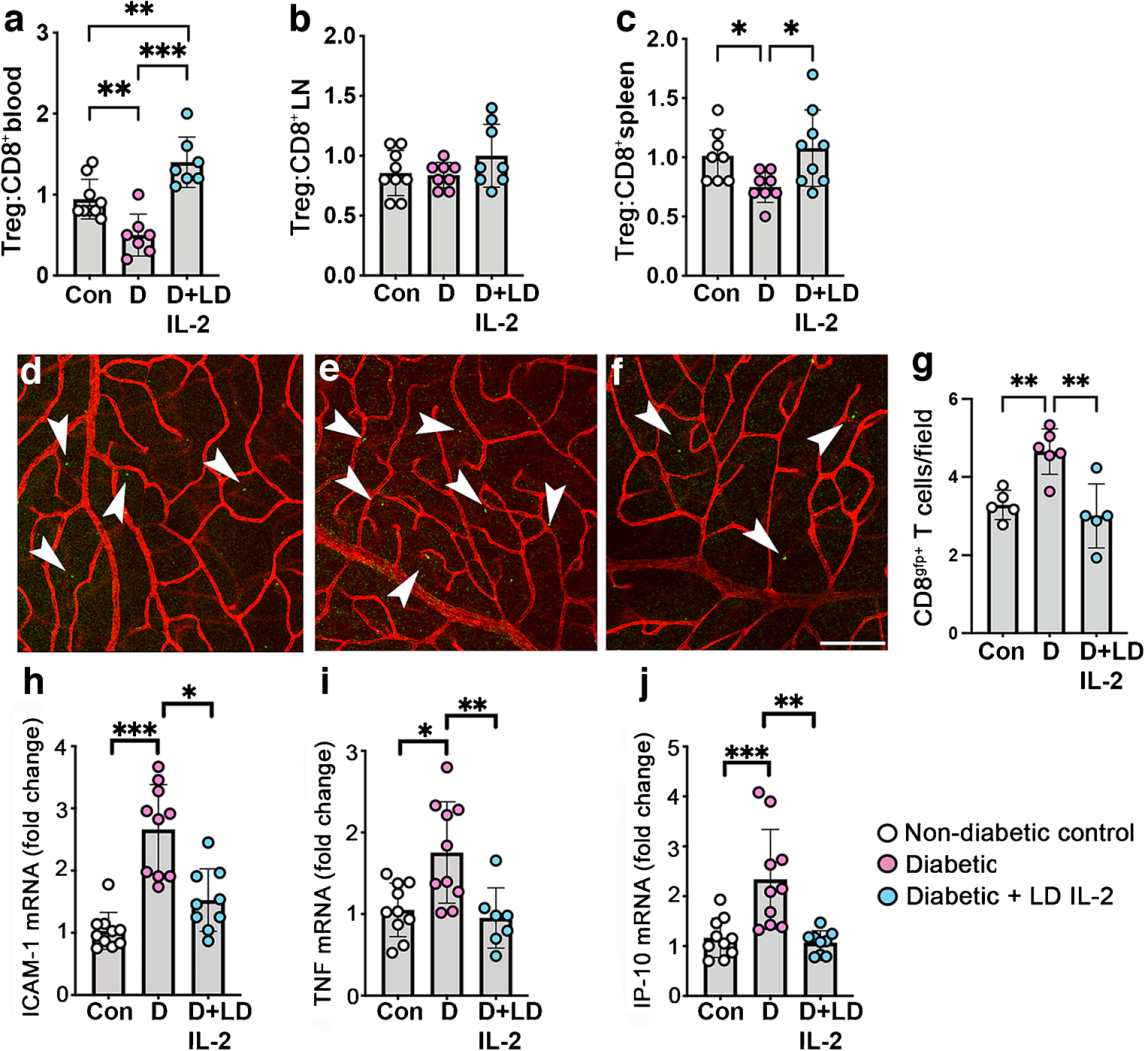
Low-dose IL-2 increased the Treg:CD8^+^ T cell ratio and reduced CD8^+^ T cells and inflammation in retinas of mice with diabetic retinopathy for 26 weeks. (**a**–**c**) Ratio of Tregs:CD8^+^ T cells in the (**a**) blood, (**b**) pooled lymph nodes (LN) and (**c**) spleen, measured by flow cytometry; *n*=7–9 mice per group from two independent experiments. (**d**–**f**) Representative flat mounts of retinas from CD8^GFP+/−^ mice, stained using DyLight 594/isolectin-conjugated isolectin B4 to show the vasculature (red): (**d**) non-diabetic control; (**e**) diabetic mice; (**f**) diabetic mice treated with low-dose IL-2. Arrowheads show CD8^+^ T cells (green). Scale bar = 100 µm. (**g**) Quantification of CD8^+^ T cells per field of retina (inner limiting membrane to outer nuclear layer); *n*=4–6 mice per group. (**h**–**j**) Fold change in mRNA levels in retinas for (**h**) ICAM-1, (**i**) TNF and (**j**) IP-10 compared with non-diabetic controls; *n*=7–10 mice per group. Con, non-diabetic control; D, diabetic mice; LD, low-dose. All values are means ± SD. All data were analysed using the Kruskal–Wallis test followed by Dunn’s test, except for the data in (**a**), (**b**) and (**g**), which were assessed using one-way ANOVA followed by the Holm–Šídák test. *p<0.05, **p<0.01, ***p<0.001

At 26 weeks of diabetes, the Treg:CD8^+^ T cell ratio in blood and spleen was reduced but remained unaltered in pooled lymph nodes compared with non-diabetic controls (Fig. 7a–c). Low-dose IL-2 increased the ratio in blood and spleen, but not in pooled lymph nodes compared with untreated diabetic mice (Fig. 7a–c). Similarly, in the retina of diabetic mice, low-dose IL-2 increased Tregs (Fig. 6) and reduced CD8^+^ T cells compared with untreated diabetic mice (untreated diabetes: 4.65 ± 0.58 vs diabetes + low-dose IL-2: 3.00 ± 0.81 cells per field, *p*<0.01) (Fig. 7d–g). ICAM-1, TNF and IP-10 mRNA levels were increased in diabetic mice compared with non-diabetic controls (Fig. 7h–j) and were reduced with low-dose IL-2 treatment compared with untreated diabetic mice (Fig. 7h–j).

### Low-dose IL-2 reduced retinal vascular pathology in diabetic retinopathy

**Fig. 8:**
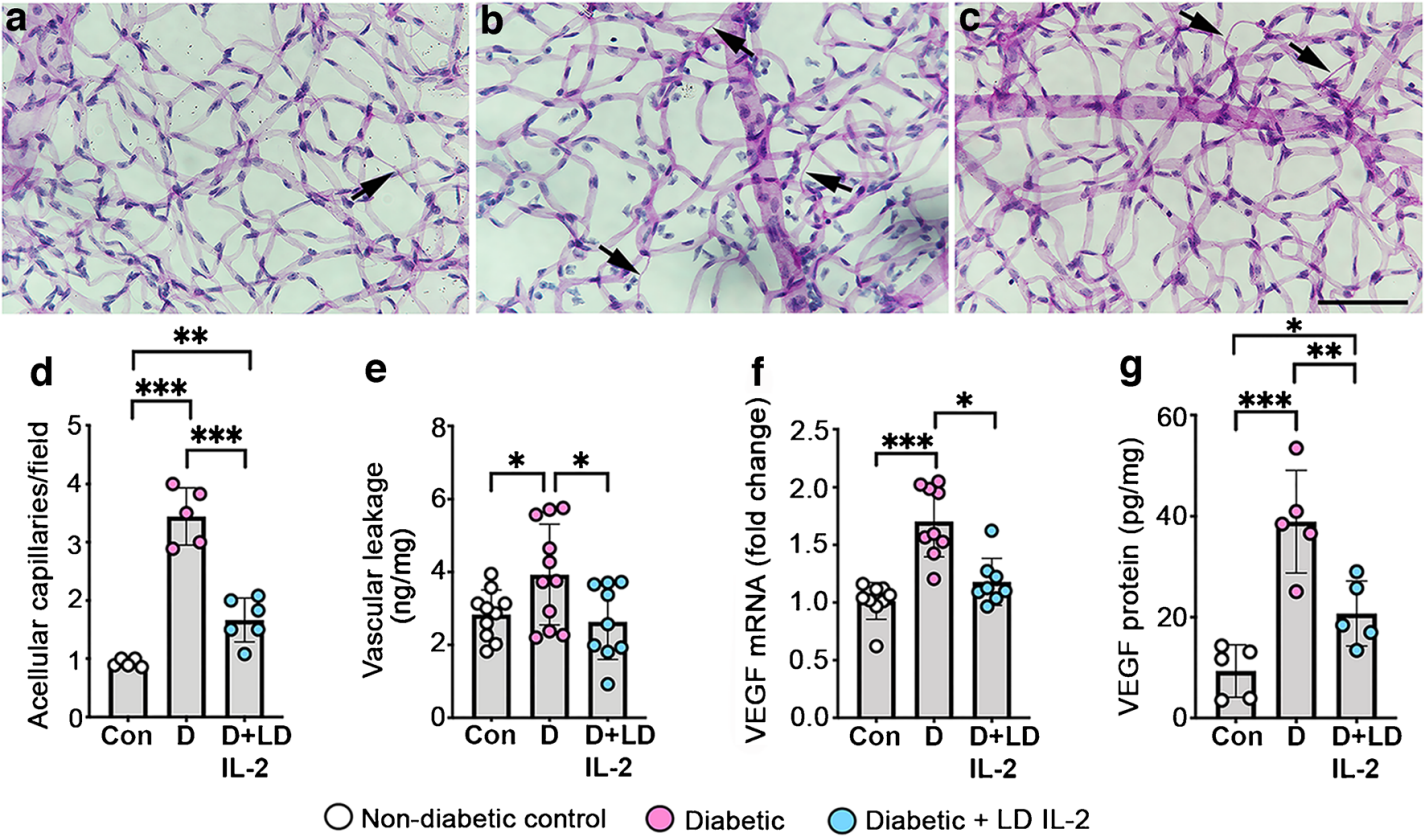
Low-dose IL2 reduced the number of acellular capillaries, vascular leakage and VEGF in retina of mice with diabetic retinopathy for 26 weeks. (**a**–**c**) Trypsin digests of retina showing the vasculature. Sections were stained using periodic acid/Schiff reagent: (**a**) non-diabetic control; (**b**) diabetic mice; (**c**) diabetic mice treated with low-dose IL2. Arrows indicate acellular capillaries. Scale bar = 100 µm. (**d**) Quantification of acellular capillaries in retina; *n*=5–6 mice per group. (**e**) Vascular leakage into the vitreous humour measured by an albumin ELISA; *n*=9–11 mice per group. (**f**) Retinal VEGF mRNA fold change compared with non-diabetic controls; *n*=8–11 mice per group. (**g**) VEGF protein in retina measured by ELISA; *n*=5 mice per group. Con, non-diabetic control; D, diabetic mice; LD, low-dose. All values are means ± SD. All data were analysed by one-way ANOVA followed by the Holm–Šídák test, except data in (**f**), which were analysed using Kruskal–Wallis and Dunn’s tests. *p<0.05, **p<0.01, ***p<0.001

Untreated diabetic mice had more acellular capillaries in the retina than non-diabetic controls, and increased vascular leakage and VEGF mRNA and protein expression (Fig. 8). Low-dose IL-2 reduced the number of acellular capillaries (0.48-fold decrease) and VEGF protein levels in the retina (albeit not to non-diabetic control levels), and reduced retinal vascular leakage and VEGF mRNA to non-diabetic control levels (Fig. 8).

## Discussion

The therapeutic benefit of low-dose IL-2 is predicated on Tregs migrating into tissues to dampen local inflammation and cellular damage [26]. The ability of Tregs to penetrate retinal tissue in neovascular retinopathy was established by our group in OIR mice [4]. The adoptive transfer of Tregs in OIR mice increased their abundance in blood and lymphoid organs and trafficking into the retina, where they protected against severe vasculopathy [4]. Here, we report that low-dose IL-2 therapy increased Tregs and reduced CD8^+^ T cells in the retinas of OIR and diabetic retinopathy mouse models. Of potential clinical relevance is the ability of low-dose IL-2 to reduce damage to the retinal vasculature, including vascular leakage, acellular capillaries, neovascularisation and upregulation of VEGF.

Our study provides insights into the dysregulation of Tregs in OIR and diabetic retinopathy. Regarding OIR, there is limited information about Tregs in the clinically relevant condition (retinopathy of prematurity). Our previous study in OIR control mice demonstrated that Tregs are present in small numbers in blood, lymphoid tissues and retina, except in acute ischaemia at P13, which likely occurs in an attempt to quench the proinflammatory environment of the retina [4]. In terms of increasing Tregs with IL-2 treatment, high-dose IL-2 had no effect, whereas low-dose IL-2 increased Tregs in blood, spleen and retina. In diabetes, peripheral blood from individuals with type 1 diabetes has a similar frequency of Tregs to that in healthy individuals without diabetes [27]. The findings of the present study are in general agreement with these findings in individuals with diabetes [27] with respect to pooled lymph nodes and spleen after 26 weeks of diabetes compared with controls, although diabetic mice had fewer Tregs in blood. However, Tregs were increased in retinal tissue of untreated diabetic mice, suggesting that, prior to the 26 week time point, Tregs were recruited into the retina to reduce inflammation but had little effect. The ability of low-dose IL-2 to increase Tregs in blood, spleen and retina of diabetic mice emphasises the utility of this approach to influence Tregs in diabetic retinopathy.

Comparing the effect of low and high-dose IL-2 on immune cells in blood and lymphoid tissues provided insight into the different actions of these treatments. Although diabetic retinopathy was not studied, the findings in OIR mice demonstrate that high-dose IL-2, but not low-dose IL-2, increased NK cells in blood compared with OIR controls, and CD8^+^ T cells in blood and NKT cells in pooled lymph nodes compared with room air controls. These data are consistent with prior studies in other diseases, and highlight the potential deleterious effects of high-dose IL-2 [7, 11, 12].

Although circulating Tregs are present in similar numbers in type 1 diabetic and healthy individuals, they have reduced functionality in individuals with type 1 diabetes [27, 28]. The preservation or bolstering of Treg activity is key to inhibiting inflammation and cell damage [29]. Low-dose IL-2 enhances the suppressive function of Tregs by upregulating immunomodulatory molecules, including CTLA-4 and PD-1 [28, 30]. In our study, Tregs expanded by low-dose IL-2 treatment exhibited increased CLTA-4 and PD-1 in the blood and peripheral lymphoid tissues of mice with OIR or diabetic retinopathy, findings that are consistent with studies of murine atherosclerosis [31] and colitis [32]. We found that low-dose IL-2 restored the number of TIGIT^+^ Tregs in diabetic retinopathy. Recent research indicates that TIGIT signalling resulted in the expression of Foxp3 in Tregs, and that upregulation of TIGIT correlated positively with CLTA-4 and PD-1 expression [33]. These findings highlight the importance of TIGIT in maintaining the functionality and stability of Tregs and the potential of boosting the specific suppressive function of Tregs to confer retinal protection in diabetic retinopathy.

A main mechanism by which Tregs reduce inflammation is the suppression of effector T cells, such as CD8^+^ T cells [8, 9], with the imbalance favouring effector T cells impacting the progression of inflammatory diseases including diabetes [26]. In our previous study of OIR control mice, CD8^+^ T cells were expanded in lymphoid tissues and trafficked into the retina via CXCR3 (C-X-C motif chemokine ligand receptor 3) and the chemokine IP-10, and released inflammatory mediators such as TNF to induce vascular injury [6]. In individuals with diabetic retinopathy, CD8^+^ T cells are present in the vitreous humour [34, 35], but their role in diabetic retinopathy has not been extensively explored. Here, we established that CD8^+^ T cells are elevated in the retina of diabetic mice compared with controls. Our finding that low-dose IL-2 increased the Treg:CD8^+^ T cell ratio in the blood and spleen of OIR mice and mice with diabetic retinopathy indicates an interaction between these cell populations. Although this interaction between Tregs and CD8^+^ T cells is not fully understood in retinopathy, the increased CTLA-4 in Tregs can suppress CD8^+^ T cells via antigen-presenting cells [36]. This may help to explain the decrease in CD8^+^ T cells in our study, and the potential of CTLA-4 as an anti-inflammatory mediator through Treg activity.

A key step in the inflammation process is adhesion of immune cells to the vasculature due to increased expression of adhesion molecules such as ICAM-1 [16, 22, 24]. Increased ICAM-1 levels in the retina occur via mechanisms including the upregulation of TNF [37]. TNF is of importance in neovascular retinopathies due to its elevated levels in the vitreous humour [38] and serum [39] of individuals with proliferative diabetic retinopathy, and its causal role in the development of OIR and diabetic retinopathy in rodents [40, 41]. Our data indicate that low-dose IL-2 decreased both ICAM-1 and TNF in the retina. We did not directly define the mechanisms involved, but speculate that Tregs reduce CD8^+^ T cells, which are a source of TNF, IFNγ and cytotoxic factors [6]. Indeed, we have reported that the adoptive transfer of CD8^+^ T cells that are deficient in TNF into Rag1^−/−^ mice ameliorated retinal inflammation and vasculopathy in OIR mice [6]. This contrasts with the retinal pathology that occurred in Rag1^−/−^ mice that were administered wild-type T cells possessing functional TNF [6]. An outstanding question is how CD8^+^ T cells are trafficked into the retina in diabetic retinopathy. The chemokine IP-10 (also known as C-X-C motif chemokine ligand 10) and its receptor, CXCR3, which is preferentially expressed by activated CD8^+^ T cells, participate in the migration of CD8^+^ T cells into inflamed tissue [42]. We previously showed that CXCR3^+^CD8^+^ T cells are increased in OIR retinas, and CXCR3 blockade reduces CD8^+^ T cells [6]. In the present study, IP-10 was increased in the retina of mice with diabetic retinopathy, suggesting that the CXCR3/IP-10 pathway is involved in CD8^+^ T cell recruitment, although this requires confirmation.

In conclusion, we demonstrate that Tregs and CD8^+^ T cells penetrate retinal tissue in diabetic retinopathy, and low-dose IL-2 increases the Treg:CD8^+^ T cell ratio and ameliorates vascular damage, indicating its potential as a treatment for diabetic retinopathy. Further studies are required to determine whether low-dose IL-2 directly protects the blood–retinal barrier and its tight junctions, and thereby prevents entry of CD8^+^ T cells into the retina. A limitation is that we did not determine the ocular levels of IL-2, which may influence the retina via its proinflammatory actions [43]. In individuals with non-proliferative and proliferative diabetic retinopathy, IL-2 levels are unchanged or reduced in vitreous and aqueous fluids compared with individuals without retinopathy [44–46]. However, IL-2 levels in the vitreous humour have been reported to be increased in individuals with diabetic retinopathy, although the study size was limited in this study [47]. It will be important in future clinical research to determine whether low-dose IL-2 increases the ocular levels of IL-2 in those with diabetic retinopathy, although there was no detrimental effect on the mouse retina. Another consideration is the finding in individuals with diabetes that, although low-dose IL-2 expands the Treg compartment, the numbers of NK and CD8^+^ T cells are increased [15]. Nevertheless, low-dose IL-2 is well tolerated in individuals with diabetes, with side-effects being limited to infection at the injection site [15, 48]. In the context of diabetes, our data, although not statistically significant, suggest that low-dose IL-2 tended to reduce HbA_1c_ levels. Treatment with a low-dose IL-2/CD25 fusion protein has been reported to preserve beta cell function and prevent diabetes progression in mice [49]. It would be interesting to consider the direct effect of low-dose IL-2 on glucose metabolism and whether engineering of IL-2 to improve its action will ameliorate diabetes and its complications, including diabetic retinopathy [50].

## Supplementary Information

Below is the link to the electronic supplementary material.

ESM (PDF 1193 KB)

## Data Availability

The data from this study are available from the corresponding author on reasonable request.

## Abbreviations

CTLA-4: Cytotoxic T-lymphocyte-associated protein-4
CXCR3: C-X-C motif chemokine ligand receptor 3
Foxp3: Forkhead box protein P3
GFP: Green fluorescent protein
ICAM-1: Intercellular adhesion molecule-1
IP-10: IFNγ inducible protein-10
NK cells: Natural killer cells
NKT cells: Natural killer T cells
OIR: Oxygen-induced retinopathy
PD-1: Programmed cell death protein-1
RFP: Red fluorescent protein
Tregs: Regulatory T cells
VEGF: Vascular endothelial growth factor
