# Supplementary material for: Immunotherapy with low-dose IL-2 attenuates vascular injury in mice with diabetic and neovascular retinopathy by restoring the balance between Foxp3+ Tregs and CD8+ T cells
Source: Diabetologia. 2025 Mar 25;68(7):1559–73. doi: 10.1007/s00125-025-06412-8 (PMC12176913; doi:10.1007/s00125-025-06412-8)
Supplement: Supplementary file 1 — ESM (PDF 1193 KB) [file 125_2025_6412_MOESM1_ESM.pdf]

Electronic Supplemental Material

Immunotherapy with low dose IL-2 attenuates vascular injury in mice with diabetic and neovascular retinopathy by restoring the balance between $Foxp3^+$ Tregs and $CD8^+$ T cells

Devy Deliyanti D, Varaporn Suphapimol, Amit Joglekar, Abhirup Jayasimhan and Jennifer L. Wilkinson-Berka

Department of Anatomy and Physiology, School of Biomedical Sciences, The University of Melbourne, Parkville, Victoria, Australia.

Address for Correspondence:

Professor Jennifer L. Wilkinson-Berka

School of Biomedical Sciences, The University of Melbourne

Level 2, Medical Building 181, Grattan Street, Parkville, Victoria, Australia, 3010

Email: jennifer.wilkinsonberka@unimelb.edu.au

ESM Table 1 Body weight of mice with oxygen-induced retinopathy.

Groups	Bodyweight (g)	Age	*n*
**$Foxp3^{rfp+/+}$**			
Room air control	7.95 ± 0.81	P18	24
OIR control	6.89 ± 0.57**	P18	22
OIR + low dose IL-2	6.72 ± 1.41**	P18	23
OIR + high dose IL-2	6.21 ± 0.69***, #	P18	22
**$CD8^{gfp+/-}$**			
Room air control	7.42 ± 0.17	P15	5
OIR control	6.45± 0.55***	P15	4
OIR + low dose IL-2	6.08 ± 0.31***	P15	6

OIR, oxygen-induced retinopathy. P, postnatal day. **$p<0.01$ and ***$p<0.001$ vs room air control. #$p<0.05$ vs OIR control. For $Foxp3^{rfp+/+}$ mice, the Kruskal-Wallis test followed by Dunn's post hoc test was used. For $CD8^{gfp+/-}$ mice, one-way ANOVA followed by Holm-Sidak's multiple comparisons test was performed. Values are mean ± SD.

ESM Table 2 Body weight, blood glucose and HbA1c levels of mice diabetic for 26 weeks.

**Groups**	**Body weight (g)**	**Blood glucose (mmol/L)**	**HbA1c (%)**	**HbA1c (mmol/mol)**	***n***
**$Foxp3^{rfp+/+}$**					
Non-diabetic control	35.09 ± 4.36	5.19 ± 1.58	4.75 ± 0.86	27.80 ± 8.83	20
Diabetic	26.82 ± 3.00***	23.26 ± 6.06***	12.3 ± 1.68***	110.5 ± 16.64***	21
Diabetic + low dose IL-2	25.4 ± 4.97***	24.79 ± 8.18***	11.2 ± 2.72***	100.0 ± 29.77***	20
**$CD8^{gfp+/-}$**					
Non-diabetic control	31.0 ± 2.91	5.68 ± 1.18	4.57 ± 0.80	16.83 ± 3.81	5
Diabetic	26.82 ± 1.90***	24.16 ± 7.70**	11.13 ± 1.75**	98.50 ± 19.22**	6
Diabetic + low dose IL-2	25.4 ± 2.30***	23.96 ± 10.26*	9.22 ± 1.39*	80.67 ± 15.86*	5

**p*<0.05, ***p*<0.01, and ****p*<0.001 vs non-diabetic control. Body weight data of all mice were analysed using a one-way ANOVA followed by the Holm-Sidak's multiple comparisons test. Blood glucose and HbA1c data of all mice were analysed using the Kruskal-Wallis test followed by Dunn's post hoc test. Values are mean ± SD.

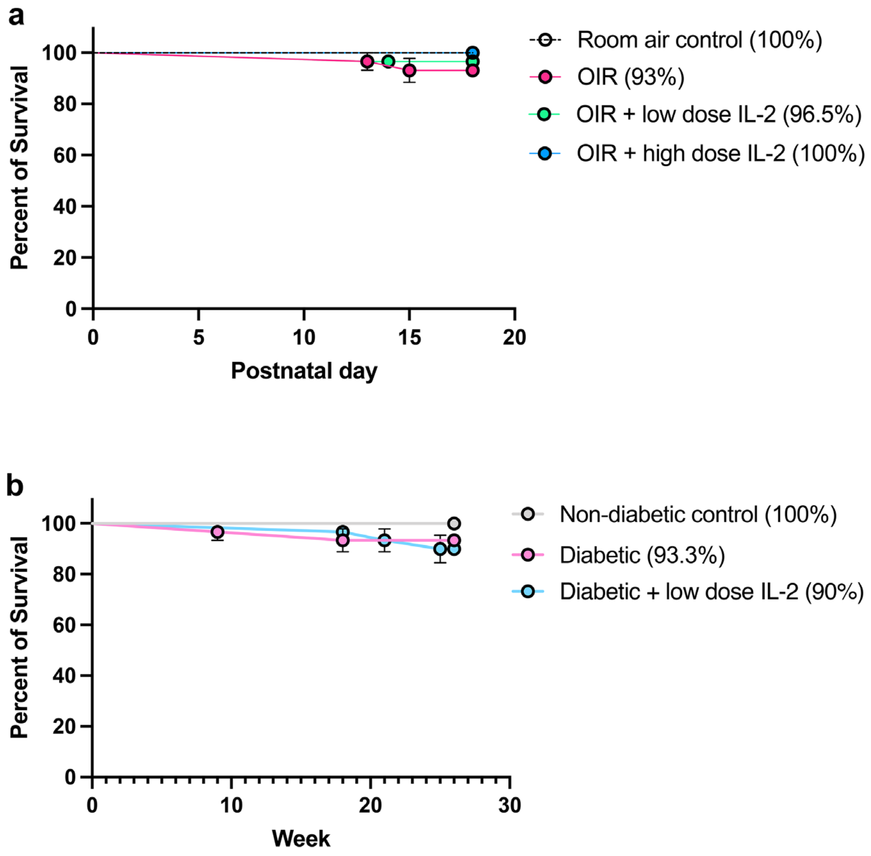

ESM Fig. 1 The effect of low dose IL-2 treatment on the survival of OIR mice and mice with diabetes. (**a**) Kaplan-Meier survival analysis for room air control, oxygen-induced retinopathy (OIR) mice and OIR mice treated with either low-dose IL-2 or high-dose IL-2. *n*, 22 to 31 mice per group. (**b**) Kaplan-Meier survival analysis for non-diabetic, diabetic mice and diabetic mice treated with low-dose IL-2. *n*, 25 to 28 mice per group. The percentages indicate survival rates at the study endpoint. No statistically significant differences were observed between groups (Log-rank Mantel-Cox test). Values are mean ± SD.

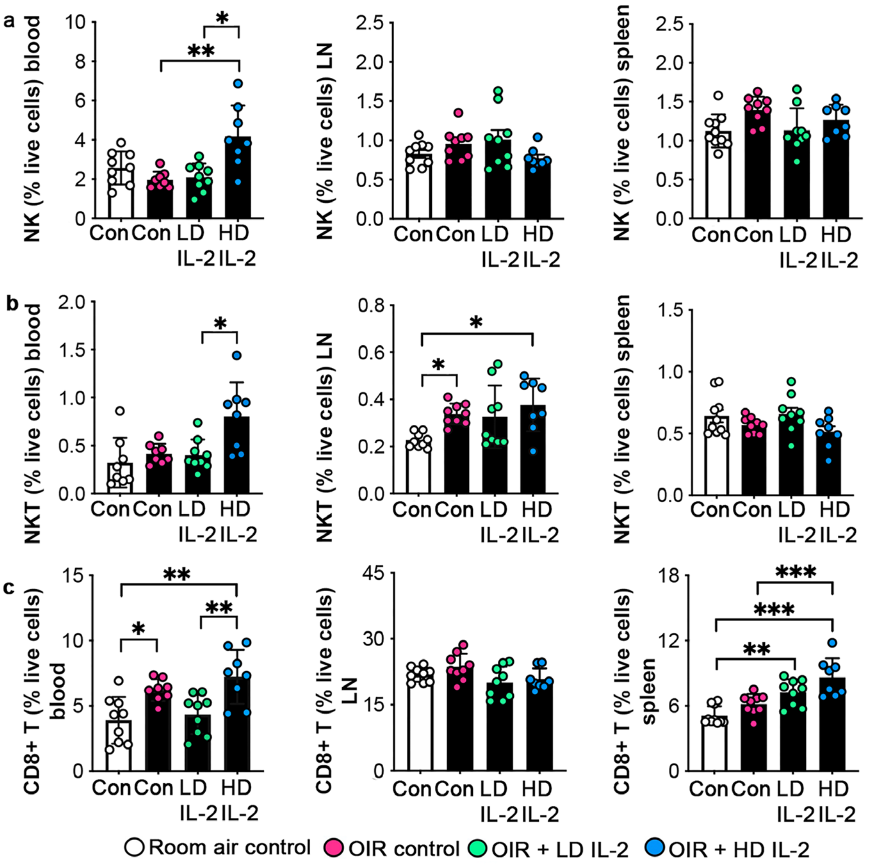

ESM Fig. 2 The effect of low dose and high dose IL-2 treatment on NK, NKT, and $CD8^+$ T cells in mice with OIR at postnatal day 18. Con, control. LD, low dose IL-2. HD, high dose IL-2. The number of (**a**) NK ($NK1.1^+CD3$-), (**b**) NKT ($NK1.1^+CD3^+$), and (**c**) $CD8^+$ T cells ($CD3^+CD8^+$) in the blood, pooled lymph nodes (LN) and spleens of control and OIR mice were measured by flow cytometry. *n,* 8 to 10 mice per group with at least two independent experiments. All data underwent analysis using the Kruskal-Wallis test followed by Dunn's test, except for the spleen data in ESM Fig. 2a and all data in ESM Fig. 2c, which were analysed using a one-way ANOVA followed by the Holm-Sidak test for non-parametric comparisons. *p<0.05, **p<0.01, and ***p<0.001. Values are mean ± SD.

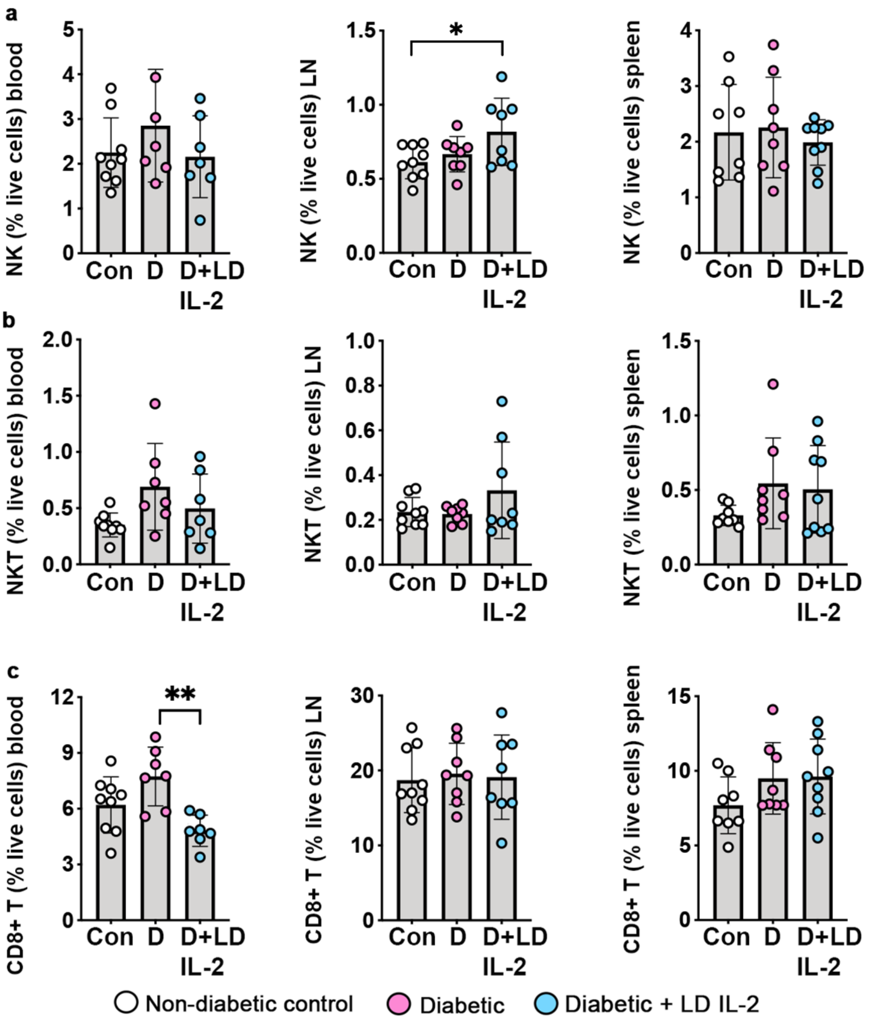

ESM Fig. 3 The effect of low dose IL-2 treatment on the abundance of NK, NKT, and $CD8^+$ T cells in diabetic mice at 26 weeks. Con, non-diabetic control. D, diabetic. LD, low dose IL-2. The number of (**a**) NK ($NK1.1^+CD3-$), (**b**) NKT ($NK1.1^+CD3^+$) and (**c**) $CD8^+$ T ($CD3^+CD8^+$) cells in the blood, pooled lymph nodes (LN), and spleens of non-diabetic and diabetic mice measured by flow cytometry. *n,* 7 to 9 mice per group. All data were analysed using a one-way ANOVA followed by the Holm-Sidak test, except for ESM Fig. 3b where the Kruskal-Wallis test was followed by the Dunn's test. **p*<0.05, and ***p*<0.01. Values are mean ± SD.
